# Research Progress on Factors Affecting Oil-Absorption Performance of Cement-Based Materials

**DOI:** 10.3390/ma16083166

**Published:** 2023-04-17

**Authors:** Dongli Wang, Siqing Liu, Bingqiang Dong, Lili Yuan, Huimin Pan, Qingxin Zhao

**Affiliations:** 1 College of Civil Engineering and Architecture, Northeast Petroleum University, No. 99 XueFu Road, Daqing 163318, China; dongli.w@126.com (D.W.); liusiqingpp@outlook.com (S.L.); 2 Key Laboratory of Green Construction and Intelligent Maintenance for Civil Engineering of Hebei Province, Yanshan University, Qinhuangdao 066004, China; hmpan2005@163.com (H.P.); zhaoqingxin@ysu.edu.cn (Q.Z.); 3 Shenzhen Guoyi Park Construction Co., Ltd., Research and Development Center, Shenzhen 518040, China; 13798592619@163.com

**Keywords:** oil-absorbing materials, oil pollutants, cement-based materials, oil-absorbing properties

## Abstract

With the wide application of petroleum resources, oil substances have polluted the environment in every link from crude oil extraction to utilization. Cement-based materials are the main materials in civil engineering, and the study of their adsorption capacity for oil pollutants can expand the scope of functional engineering applications of cement-based materials. Based on the research status of the oil-wet mechanism of different kinds of oil-absorbing materials, this paper lists the types of conventional oil-absorbing materials and introduces their application in cement-based materials while outlining the influence of different oil-absorbing materials on the oil-absorbing properties of cement-based composites. The analysis found that 10% Acronal S400F emulsion can reduce the water absorption rate of cement stone by 75% and enhance the oil-absorption rate by 62%. Adding 5% polyethylene glycol can increase the oil–water relative permeability of cement stone to 1.2. The oil-adsorption process is described by kinetic and thermodynamic equations. Two isotherm adsorption models and three adsorption kinetic models are explained, and oil-absorbing materials and adsorption models are matched. The effects of specific surface area, porosity, pore interface, material outer surface, oil-absorption strain, and pore network on the oil-absorption performance of materials are reviewed. It was found that the porosity has the greatest influence on the oil-absorbing performance. When the porosity of the oil-absorbing material increases from 72% to 91%, the oil absorption can increase to 236%. In this paper, by analyzing the research progress of factors affecting oil-absorption performance, ideas for multi-angle design of functional cement-based oil-absorbing materials can be obtained.

## 1. Introduction

With the continuous enhancement of human energy utilization level, the demand for the utilization of petroleum resources is also increasing day by day [1,2]. In the process of oil extraction, transportation, refining, and consumption, minor oil spills and frequent accidents have a huge impact on the ecological environment. For example, if oil leaks at sea, the water in near-water areas and estuary waters will be polluted by a large amount of oily sewage [3], which will then spread to fresh water bodies. Oil pollution not only destroys the local ecological environment and affects people’s quality of life but also threatens the development of tourism and the economy [4,5]. Difficult-to-degrade components of petroleum pollutants, such as hydrocarbons, can be deposited around the contaminated environment. Researchers found a large number of high-molecular-weight hydrocarbons in offshore sediments two years after the oil spill [5,6]. 

Conventional oil-absorbing materials [7] have the characteristics of large adsorption capacity and fast adsorption rate, but they do not have structural properties. Cement-based materials are widely used in slope protection projects of the Haihe River, as well as in high-pollution maintenance projects such as oil fields, refineries, and oil storage depots [8]. Improving the oil purification efficiency of cement-based materials in maintenance projects and enhancing their adsorption performance for pollutants [9,10] can improve the self-purification ability of oil polluted water environment [11]. This paper discusses the factors affecting the oil-absorption performance of traditional oil-absorbing materials and cement-based materials, analyzes the oil-adsorption process from the aspects of kinetics and thermodynamics, and matches oil-absorbing materials and adsorption models. The effects of specific surface area, porosity, and pore interface on oil-absorption performance were analyzed. The paper can provide a reference for oil-absorption modification of cement-based materials.

**Figure 1:**
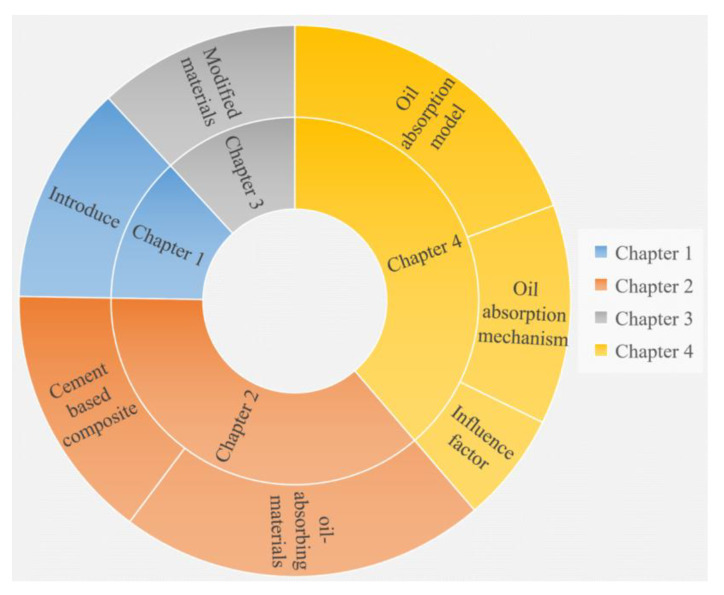
The distribution of reference articles in each section of the paper.

The keywords and search terms used in the literature review search were as follows: oil-absorbing materials, cement, concrete, oil pollutants, oil-absorbing process, oil-absorbing mechanism, and oil-absorbing models. The relevant papers are summarized, and the distribution of reference articles is shown in Figure 1.

## 2. The Influence of Oil-Absorbing Materials in Cement-Based Materials

There are many ways to deal with oil spill accidents, such as incineration [12], using materials with sorption capacity to absorb oil pollution [13], and using biosurfactants to remove oil pollution [14]. The adsorption method is favored by researchers due to its high efficiency, low secondary pollution, and flexible application scenarios [15]. Therefore, this paper will focus on the adsorption method to discuss oil-absorbing materials. The ideal oil-adsorption material should possess such characteristics as high sorption capacity, good oil–water selectivity, and convenient recovery and should be regenerable and reusable [16]. Adding high-efficiency oil-absorbing materials can help cement-based materials achieve better oil-absorbing effects [17]. There are many types of oil-absorbing materials, and each has advantages and disadvantages. Some oil-absorbing materials and their applications and effects in cement-based materials are described below.

### 2.1. Fiber Materials and Their Cement-Based Composite Materials

**Figure 2:**
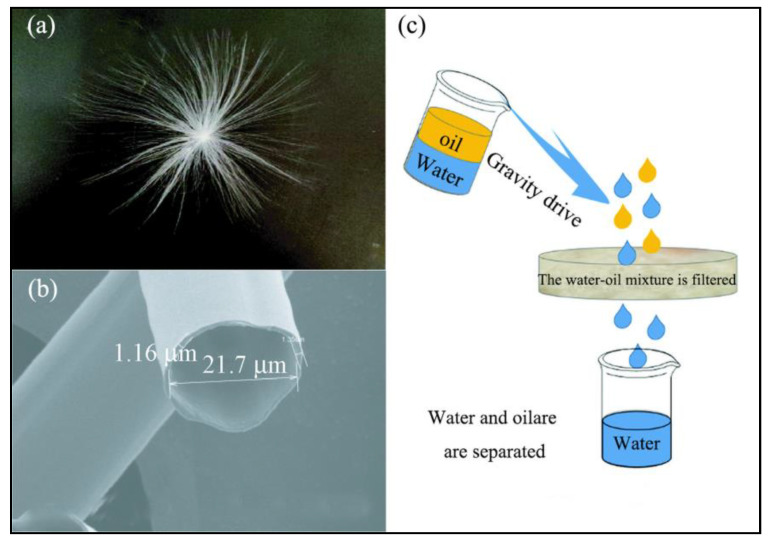
(**a**) Single fiber velvet; (**b**) cross-sectional morphology (×2000), (**c**) schematic diagram of oil–water separation [22].

Natural fiber adsorption materials are usually composed of crop wastes with large output and low utilization rate, which belong to natural organic adsorption materials. Straw [18], rice husk [19], etc. are all natural fiber adsorption materials. The surface of some plants is superhydrophobic, so these plants have the characteristics of oil–water selection without hydrophobic modification [20]. In order to find the natural plant fiber with the best comprehensive oil-absorption performance, researchers conducted oil-absorption experiments on different kinds of plant fibers [21]. Wang et al. [22] found that nature hollow metaplexis japonica seed hair fibers not only have a highly hollow structure, as shown in Figure 2b, but also have hydrophobic and lipophilic surface features. Figure 2c shows that nature hollow metaplexis japonica seed hair fibers have excellent sorption capacity for oil, as high as 81.52 g·g^−1^ for vegetable oil, and this material has high oil retention rate and reuse rate.

As a man-made fiber, polypropylene fiber has excellent hydrophobic and oil-absorbing properties. Because of its high strength, light weight, and good durability, it is widely used in the research of configuring high-performance oil-absorbing materials [23]. Chai et al. [24] discovered a new oil absorbent named polypropylene fiber-grafted poly butyl methacrylate (PP-g-PBMA). PP-g-PBMA was prepared by suspension polymerization using butyl methacrylate as a monomer, benzoyl peroxide (BPO) as an initiator, and N,N′-methylene bis acrylamide as a cross-linker. After studying the effects of monomer concentration, initiator concentration, and cross-linker dosage on oil-absorption performance, Chai et al. prepared an oil-absorbing agent with the best oil-absorption performance. Compared with the Langmuir sorption isotherm, the Freundlich sorption isotherm describes the sorption equilibrium process better. Oil-absorption experiments showed that the maximum sorption capacities of PP-g-PBMA for toluene, diesel, soybean oil, and lubricating oil could reach 18.65, 25.74, 33.56, and 38.90 g·g^−1^, respectively. In addition, PP-g-PBMA can be reused more than eight times and maintains good adsorption performance.

**Figure 3:**
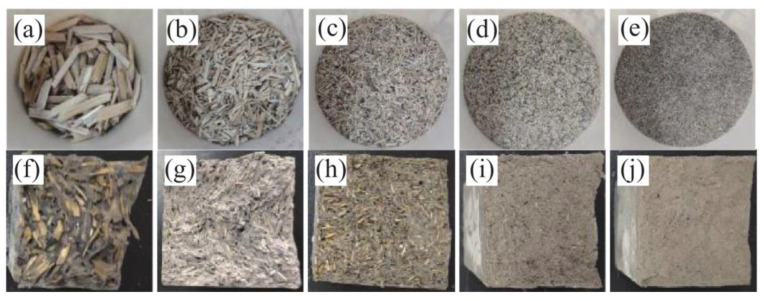
Morphology of hemp straw fiber: (**a**) 4000 μm; (**b**) 1700 μm; (**c**) 830 μm; (**d**) 380 μm; (**e**) 180 μm; and cross section of hemp straw fiber/cementitious composites with different particle sizes: (**f**) 4000 μm; (**g**) 1700 μm; (**h**) 830 μm; (**i**) 380 μm; (**j**) 180 μm; [26].

The research direction of plant-fiber-reinforced cement-based composites focuses on fiber length, surface physical properties, and the effect of its own internal structure on the toughness, impact resistance, and crack resistance of the material [25]. Wang et al. [26] studied the effect of different particle sizes of hemp straw fibers on the strength of low-content fly ash cement. Figure 3a–e show the morphology of hemp straw fiber/cementitious composites with different particle sizes, and Figure 3f–j show the cross sections of hemp straw fiber/cementitious composites with different particle sizes. When the particle size of hemp straw fiber is 1700 μm and the addition amount (mass ratio) is 12%, the natural hemp straw fiber cement-based composite has the best flexural strength and specific strength.

The use of polypropylene fibers in cement-based materials can improve the impact resistance and flexural strength of concrete [27]. Banthia N. et al. [28] studied the development of early cracks in concrete with polypropylene fibers and found that fine fibers are more effective than coarse fibers, and long fibers are more effective than short fibers in controlling concrete plastic shrinkage cracks. Incorporation of polypropylene fibers can enhance the mechanical properties of concrete, and it can also increase the durability of concrete [29].

### 2.2. Aerogel Material Cement-Based Composite Material

Aerogel materials, as a new type of oil-absorbing material, have the advantages of large specific surface area, high porosity, and low density [30]. There is a large amount of adsorption space inside aerogel, but the mechanical properties and recycling performance of aerogel oil-absorbing materials are poor [31]. Z. Rahmani et al. [32] synthesized N-doped graphene aerogel with a 3D interconnected network using graphene oxide and pyrrole in an aqueous medium with ammonia. The prepared n-doped aerogel exhibited elevated specific surface area (340 m^2^·g^−1^), hydrophobic nature, and excellent adsorption capacity (210 g·g^−1^ for crude oil removal). The recyclability of the adsorbent was also investigated, and the oil sorption capacity decreased only slightly after ten cycles of oil absorption.

Aerogel cement-based composites have many advantages, such as light weight, good heat insulation, and high fire resistance, so they are mainly used in concrete thermal insulation exterior walls and fireproof lining members. Lu et al. [33] added silane coupling agent modified aerogel slurry into a nano-silica-enhanced cement paste to prepare lightweight aerogel/cement composites (ACCs). They also investigated the effect of the modification on the pore structure and hardened performance of ACCs. The results showed that the thermal conductivity of ACCs was 0.067 w·(m·K)^−1^, the compressive strength was 1.2 MPa, and the density was 390 kg·m^−3^ when the modified aerogel slurry replaced the cement paste with a volume fraction of 66%. In addition, experiments show that the total porosity and average pore diameter of the composite are 72.8% and 170.9 nm, respectively. Nano-silica can enhance the matrix. Surface modification of aerogel results in better compatibility and facilitates the preparation of ACCs with synergistic mechanical properties and thermal insulation properties.

### 2.3. Resin Cement-Based Composites

High oil-absorption resin [34] is a resin with a 3D network cross-linked structure, and according to the main components of oil-absorbing resin, it is divided into two categories: acrylic resin [35] and olefin resin [36]. Oil-absorbing resin has many advantages such as ability to absorb a variety of oils, good hydrophobicity, and good oil retention, but it also has disadvantages such as low oil-absorption rate and poor oil-absorption reusability. In order to overcome these shortcomings, Liu et al. [37] developed a waste epoxy resin (EP)-derived hydrophobic modifier to fabricate high-efficiency oil absorbents by simple dip-coating on melamine foam (MF). The resulting high-efficiency oil absorbents exhibited excellent absorption performance for various oils and organic solvents with an ultrafast adsorption rate of 2 s and high oil sorption capacity of 116 g·g^−1^.

Resin materials are widely used in the field of cement-based materials and are mainly divided into two categories: hybrid resin materials [38] and polymer emulsions [39]. By adding water-absorbing resin to cement-based materials, the purpose of internal curing can be achieved, and finally the crack resistance of cement-based materials can be increased, and the generation of internal cracks in mass concrete can be reduced [40]. By adding polymer emulsions to cement-based materials, the strength and durability of cement-based materials can be increased [39]. Qi et al. [41] prepared oil-absorbing resin using stearyl methacrylate (SMA), butyl methacrylate (BMA), and styrene, modified it with nano-silica, and used it to prepare oil well cement. They also found that the oil-absorption rate of the modified oil-absorbing resin was increased by more than 10 times at 80 °C, and the impact resistance of the modified resin cement-based composite material was significantly improved. With the increase in resin particle size, the strength of oil well cement first increased and then decreased, and the impact resistance decreased gradually. Finally, the experiment showed that the optimal particle size of modified oil-absorbing resin was 470 μm.

### 2.4. Application of Cement-Based Materials in Oil Pollution Treatment

**Figure 4:**
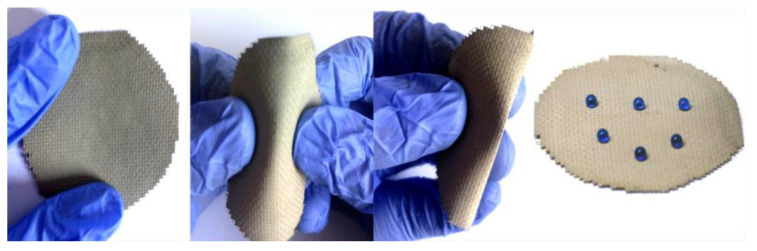
The process of bending the SOO cement-coated mesh and water droplets on the bent SOO cement-coated mesh [42].

Cement-based materials are widely used in the treatment of oily sewage. For example, the coating of cement-based materials can form a super-wettable surface structure on the network structure, so as to filter oily sewage efficiently. Li et al. [42] mixed aqueous silicane, water, and PO 42.5 into a paste with a mass ratio of 1:1:2 and immersed a Cu mesh into it to prepare a superhydrophobic–superoleophilic (SOO) cement-coated mesh. The Cu mesh can quickly absorb oil in the oil–water environment. When using this Cu mesh to filter the oil–water mixture, it can effectively prevent water from passing through (Figure 4) and separate oily substances with a separation efficiency of over 90%.

Guo et al. [43] immersed a stainless-steel mesh in the cement paste solution for 3 min and then took it out to dry. Finally, they prepared a cement stainless-steel mesh (CCSSM), which was superhydrophilic and superoleophobic underwater. When the oil–water mixture passes through the CCSSM, it can effectively prevent oil substances from passing through, and the separation efficiency exceeds 90%. In harsh environments, CCSSM can maintain good durability and corrosion resistance. The cement paste coating has excellent hydrophilicity and oleophobicity on the surface of the network structure, but the coating formed by adding silane to the cement paste has excellent hydrophobicity and lipophilicity. Substantial evidence shows that if a simple modification is made to a cement-based material, it will have two very opposite properties. 

Nikookar et al. [44] used produced water in petroleum engineering instead of mixing water, and sewage was consumed through the hydration process of cement-based materials. When the concentration of exploited water reached 60%, the late strength of cement increased by 60%, and the early hydration heat flow in the coagulation process increased by 120%. This approach allows cement-based materials to combine strength properties and stain-removing properties. Maranhão et al. [45] prepared a kind of magnetic geopolymer oil-absorbing material with an oil-absorbing capacity of 150 g·g^−1^ by using modified magnetic nanoparticles and high-altitude territory as raw materials and treating them with a pore-forming agent (H_2_O_2_) in an alkaline environment. Compared with organic oil-absorbing materials, this inorganic non-metallic material has the same oil pollution treatment effect. Moreover, the inorganic oil-absorbing material has many advantages such as a simple preparation process, low production cost, and easy acquisition of raw materials.

In summary, the oil-absorbing material and the cement-based material are both porous materials, and the oil-absorbing material can have the characteristics of good oil–water selectivity, high oil-absorption rate, and fast oil-absorption rate through appropriate modification. Its cement-based composites have excellent performance in durability, thermal insulation, mechanical properties.

## 3. Influence of Organic Matter Modification on Oil Absorption of Cement-Based Materials

For the lipophilicity of cement-based materials, most of the existing research results come from oil well cement in cement engineering. Oil well cement, a specialty cement, is commonly used to fill the annular space between production pipelines and formations, displace drilling fluids, restrict fluid movement between formations, and isolate productive and non-productive areas [46,47]. In the environment of downhole high temperature and high pressure and gaseous phase oil–water mixture, by increasing the lipophilicity and oil phase permeability of oil well cement [48], the bonding performance between the cementing structure and the oil production pipeline and the stability of the oil production process can be improved [49], and the occurrence of annular air channeling can be prevented. Therefore, an increasing number of scholars have begun to study the modification of oil well cement’s oleophilic properties, and these research results can provide some reference for the improvement of oil-absorption properties of ordinary cement-based materials.

### 3.1. Modified Materials

The methods of modifying the lipophilicity of oil well cement mainly include adding modified emulsion and adding specific admixtures [50]. The existing research on oil well cement mainly focuses on the experimental research on lipophilicity and oil phase permeability. The types and characteristics of different types of oil-well-cement-modified emulsions and their modified admixtures are summarized in Table 1.

The purpose of enhancing lipophilicity is to improve the sand and leakage prevention and impact resistance properties of oil well cement. Adding specific modified oil-absorbing materials to oil well cement can make it expand when encountering oil in the cement and have a self-healing effect on the micro-cracks of the cement, so as to prevent the hazards such as annular channeling after cementing [17]. The oil-absorption performance of modified cement is mainly affected by additives. If the number of micropores in the pore distribution of cement, the connectivity of pores, and the interface properties of cement pores are improved, the oil-absorption performance of cement can be improved [49].

### 3.2. Influence of Material Modification on Oil-Absorption Performance

Yang et al. [55] used Acronal S400F emulsion-modified oil well cement, measured the linear expansion strains of adsorbed deionized water and diesel before and after modification, and found that the linear expansion strain during the adsorption process has a linear relationship with the water absorption and oil absorption. Finally, according to the capillary permeability theory, the relationship between the saturated oil-absorption rate *A*_m_ and the linear expansion rate *ε*_w_ of the cement for diesel adsorption is obtained as follows:

(1) $$\varepsilon_{w}=\alpha \frac{2\pi \rho RT\cos^{2}\theta }{3ME}\frac{\rho }{\rho_{0}}A_{m}$$

*α* is the contribution coefficient of the pore structure to the axial expansion strain, which is taken as 1.0 when nothing is added to the cement, and 0.9 for the modified cement; *R* is the gas constant, *R* = 8.314 J·(mol·K)^−1^; *T* is the thermodynamic temperature; *θ* is the contact angle between the medium and the cement stone; *ρ*_0_ = 1000 kg·m^−3^; *M* is the molar mass of water, *M* = 18.02 g·mol^−1^; *E* is the elastic modulus of cement; and *A*_m_ is the water or oil-absorption rate of the saturated mass of cement. 

The experiment found that the saturated water absorption rate of unmodified oil well cement is 40%, while the saturated oil-absorption rate is 25%, and the results show that there are many small pores in the oil well cement, which cannot be filled with oil medium. Since small pores cannot absorb oil, the saturated oil-absorption strain is significantly smaller than the saturated water absorption strain. The saturated oil-absorption rate of modified oil well cement increased slightly from 9% before modification to 10.5%, with a growth rate of 17%. The oil-absorption swelling strain increased by 40% compared with that before modification.

Ding et al. [54] studied polyethylene glycol (PEG)-modified selectively permeable oil well cement based on the principle of electric double layer and wettability. It was found that when 5% PEG was added, the pores in the cement stone changed from the original water-wet to oil-wet. The flow of water in the oil-wet pores is blocked, so the water phase permeability decreases by 41%; on the contrary, the oil phase permeability increases by 97%, and the ratio of oil–water permeability reaches 1.2. The selective permeability of cement stone is significantly improved after the addition of PEG. Wang et al. [53] added oil-soluble resin into oil well cement to prepare a kind of selectively permeable cement stone. Oil-soluble resin has 89% solubility in diesel oil but only 2% solubility in water. With the addition of 15% oil-soluble resin, the thickening time increased from 310 to 330 min, and the compressive strength decreased from 6.5 to 5.2 MPa. During the 48 h permeability test, the ratio of relative permeability between oil and water increased from 3.0 to 7.1. Therefore, adding resin materials to cement-based materials as a permeability enhancer can significantly improve the oil phase permeability of cement stones [56].

Adding different kinds of modified materials has different effects on concrete. Porous medium oil-absorbing materials generally have great deformation ability, which increases the impact resistance of cement to varying degrees. Adding modified emulsions can improve the oil-absorbing and hydrophobic capacity of oil well cement. In order to obtain composite concrete with different oil-absorption properties, research on many aspects is still required.

## 4. Principles and Influencing Factors of Oil Adsorption

### 4.1. Oil-Adsorption Process

**Figure 5:**
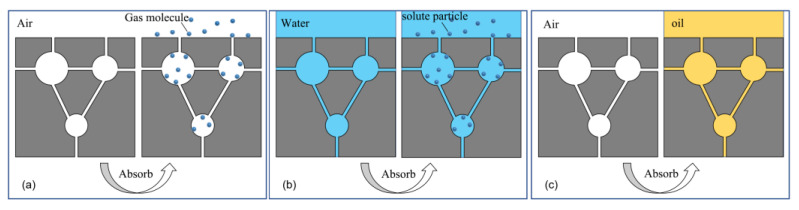
Three general systems in adsorption process: (**a**) gas adsorption; (**b**) solute adsorption; (**c**) liquid phase adsorption.

The adsorption process of porous media refers to the phenomenon that some components in the interface layer are enriched, while the other components are excluded [57]. By definition, there are three general systems for adsorption [58,59]: (a) adsorption of another gas in the gaseous phase pore space; (b) adsorption of solutes in liquid-phase pore space; (c) adsorption of liquid in gaseous phase pore space. Figure 5c shows the adsorption system of oil-absorbing materials to oil substances under normal circumstances, the gas phase components at the pore interface in the oil-absorbing material are enriched and expelled by the oil in the liquid phase, and then the formed oil substances occupy the pore space, which is the process of oil adsorption [60]. In the process of cement oil absorption, cement acts as a porous medium adsorbent, and oil acts as an adsorbate. After a period of adsorption, the gas phase in the pore space is completely replaced by the oil phase, and the oil-absorbing material gradually becomes saturated with oil [61].

Molecular dynamics indicates that the reason for the adsorption is the imbalance of atomic or molecular forcefields on the surface of the object [62]. Due to the unbalanced force, the interfacial molecules on the solid surface can reach the mechanical equilibrium of the surface by adsorbing gas or liquid molecules. This adsorption is usually in the form of physical adsorption, intermolecular through the influence of van der Waals force, and finally reach an equilibrium state of adsorption [63].

In terms of thermodynamics, the state of the system is determined by the state Gibbs function [64], as in Equation (2),

(2) G=U−TS+pV=H−TS

In the equation, *U* is the internal energy of the system, *T* is the temperature (thermodynamic temperature, *K*), *S* is entropy, *p* is the pressure, *V* is the volume, and *H* is enthalpy. *G* is also known as free enthalpy or Gibbs free energy, and the unit is J, also known as the Gibbs function of a solid surface. The change in free energy determines whether the adsorption can proceed spontaneously [65]. If the free energy decreases after adsorption, it is spontaneous adsorption, for example, the surface free energy of the hydrophilic and lipophilic material decreases when it encounters water or oil, and the adsorption proceeds spontaneously; for hydrophobic and oleophilic materials, water cannot wet the surface of the materials. Because water increases the surface energy, the adsorption cannot proceed spontaneously. However, if water is non-wetting on the surface of the materials, oil is adsorbed spontaneously [66]. 

The macroscopic performance of adsorption kinetics [67] involve the liquid phase diffusing inside the porous oil-absorbing material through capillary force; the macroscopic performance of adsorption thermodynamics [68] refers to the wettability of the oil-absorbing material interface and the spontaneous adsorption behavior of the oil-phase interface on the lipophilic interface. The process of oil substances being adsorbed by porous media oil-absorbing materials is as follows: (1) oil molecules are in contact with the surface of the porous medium, and the wetting rate is affected by the rate of change of the contact angle *θ*; (2) after the surface is wetted, the oil phase liquid further diffuses through the surface pores of the porous medium and enters the internal macroporous area; (3) after the macropores reach a certain saturation, they diffuse into the mesopore and micropore regions through the macropores; (4) oil molecules interact with the pore interface, resulting in effective physical adsorption.

### 4.2. Common Models for Adsorption Process Analysis

The adsorption model describes the adsorption process well and evaluates the adsorption capacity of the adsorbent. There are five commonly used adsorption models for the adsorption of oil pollutants: the Langmuir isotherm model [69], Freundlich isotherm model [70], pseudo-first-order (PFO) model [71], pseudo-second-order (PSO) model [72], and internal diffusion model. The adsorption isotherm model and the adsorption kinetic model can describe the interaction mechanism between the adsorbent and the adsorbate in the constant temperature and constant pressure environment. The Langmuir adsorption isotherm model, originally developed to describe gas–solid-phase adsorption onto activated carbon as an empirical model, assumes that the adsorbed layer is one molecule in thickness (monolayer adsorption), whereby the adsorption process occurs on a homogeneous adsorption site, a single molecule is adsorbed at a single site, and finally a monolayer adsorption equilibrium is reached [73].

The Freundlich adsorption isotherm model can describe reversible and nonideal adsorption processes. In contrast to the Langmuir isotherm model, the Freundlich model is not limited to monolayer adsorption but is extended to multilayer adsorption, where a single adsorption site can form hierarchical multi-molecular adsorption. During the adsorption process, the adsorption heat and affinity at the interface are non-uniformly distributed [74]. The linear formula of Langmuir isotherm adsorption and the linear formula of the Freundlich adsorption isotherm model are given in Formulas (3) and (4):

(3) $$\frac{C_{e}}{q_{e}}=\frac{1}{q_{\max}K_{L}}+\frac{C_{e}}{q_{\max}}$$

(4) $$\log q_{e}=\log k_{F}+\frac{1}{n}\log C_{e}$$

where *C_e_* is the concentration of oil pollutants in the solution, mg·L^−1^; *q_e_* and *q*_max_ are the adsorption capacity and the saturated adsorption capacity at adsorption equilibrium, mg·g^−1^; *K_L_* and *K_F_* are the parameters of the Langmuir and Freundlich models, respectively, and the unit of *K_L_* is L·mg^−1^; and 1/*n* is the intensity of the adsorption or surface heterogeneity indicating the energy relative distribution and the adsorbate sites’ heterogeneity. When 1/*n* is greater than zero (0 < 1/*n* < 1), the adsorption is favorable; when 1/*n* is greater than 1, the adsorption process is unfavorable, and it is irreversible when 1/*n* = 1 [75].

The adsorption kinetic models are mainly used to evaluate the performance of the adsorbent and to investigate the adsorption mass transfer mechanisms [76]; among them, the PFO models and the PSO models are most used in the adsorption process of oil pollutants. The formulas of the models are as follows in (5) and (6):

(5) $$\ln\left(q_{e}-q_{t}\right)=\ln q_{e}-k_{1}t,$$

(6) $$\frac{t}{q_{t}}=\frac{1}{k_{2}\cdot q_{e}^{2}}+\frac{t}{q_{e}}$$

*k*_1_ is the pseudo-first-order rate constant, L·min^−1^; *k*_2_ is the pseudo-second-order rate constant, g∙mg^−1^∙min^−1^; *q_t_* is the adsorbed amount of the adsorbate at time *t*, mg∙g^−1^; and *q_e_* is the adsorption capacity at equilibrium, mg∙g^−1^.

In order to better describe the process of adsorbate diffusion from adsorbent surface to the interior, the researchers used the Weber and Morris model (intraparticle diffusion model) to further explain the process of oil diffusion and mass transfer inside the oil-absorbing material [67], as shown in Formula (7):

(7) $$q_{t}=k_{d}\cdot t^{1/2}+I$$

*k_d_* is the intraparticle diffusion rate constant, mg·(g·s^1/2^)^−1^; *I* is the intercept. Depending on the oil-absorption rate, the phase curves of liquid film diffusion and intraparticle diffusion can be monitored. Depending on the oil-absorption rate, the isophase curves of liquid film diffusion and intraparticle diffusion can be monitored.

Cai et al. [77] measured the adsorption curve of crab-shell-activated biochar to diesel oil through experiments. Through the isotherm model fitting, it was found that the adsorption process is more in line with the Freundlich isotherm adsorption model. The adsorption of diesel oil on the pore interface of crab-shell-activated biochar is the result of multi-layer adsorption, and non-uniform surfaces and pores are the main adsorption sites. Sarkheil et al. [78] fit the adsorption curve of bagasse to adsorb petroleum pollutants, and the results show that compared with the Freundlich isotherm model, the Langmuir isotherm model is more suitable for describing the adsorption process.

Singh et al. [79] prepared a nanocomposite of Fe_3_O_4_/chitosan, a superparamagnetic oil-absorbing material. Based on the data obtained from the diesel adsorption experiment, the adsorption model was fitted to the material, and the Langmuir isotherm model and the pseudo-second-order model were well fitted. Abel et al. [80] found that the adsorption of crude oil from water by coconut-coir-activated carbon conformed to the Freundlich isotherm adsorption model and the pseudo-second-order model. Davoodi [81] conducted experiments on the removal of oil stains from water by hydrophobic dolomite, and the results showed that the pseudo-second-order model could better describe the adsorption process. Wang [82] carried out adsorption experiments of rice husk cellulose on diesel and corn oil, and the results showed that the pseudo-second-order adsorption model and the Freundlich model fit the adsorption curve. 

In Guo’s [83] study, the oil-absorption kinetic model and isotherm adsorption model of graphene/chitosan composite aerogel microspheres fit the pseudo-second-order model and the Langmuir model, respectively. Khalifa [84] studied the adsorption performance of newspaper/polystyrene composite adsorbent on petroleum pollutants and found that it conformed to the Langmuir model and the pseudo-second-order model. Akpomie [85] studied the treatment of light crude sewage by rice husk montmorillonite composites and found that the pseudo-first-order model can describe the adsorption process well. Liu [86] studied the adsorption curve of methyl trimethoxy silane coated loofah–chitin aerogel to benzene series, which is more in line with the Langmuir isotherm model and the pseudo-first-order model.

When the isotherm adsorption model [75] is fitted to the adsorbate concentration and adsorbent adsorption capacity, it is particularly important to detect the concentration of oil in water. If it is difficult to detect the concentration of adsorbate, a kinetic model can be used for fitting. By monitoring the adsorption capacity, equilibrium adsorption capacity, and time, etc. and then fitting the experimental data, the kinetic adsorption model [76] that conforms to the adsorption process can be finally determined. For example, for the adsorption system of type (c) in Figure 5, it is more appropriate to select the kinetic model for analysis. According to the different adsorption systems and the difficulty and accuracy of obtaining the corresponding physical quantities, selecting an appropriate model for fitting analysis can more fully explain the adsorption mechanism of oil-absorbing materials [57].

### 4.3. Influence of Physical and Chemical Properties of Oil-Absorbing Materials on Oil-Absorbing Performance

Oil-absorbing materials and cement-based materials are both porous media adsorption materials. By studying the adsorption mechanism and design ideas of oil-absorbing materials, the adsorption performance of cement-based materials for oil pollutants can be improved. Through combing the research on the design and adsorption mechanism of oil-absorbing materials, it was found that the porosity, specific surface area, pore interface, outer surface, oil-absorbing strain, and pore network of the material have a direct impact on all aspects of oil-absorbing performance. The author summarizes the effects of the physical and chemical properties of the existing oil-absorbing materials on the oil-absorption rate, oil absorption, lipophilicity, etc., which are summarized in Table 2.

A comprehensive comparison of factors affecting oil-absorption performance found that porosity had the greatest impact on oil absorption. Yu et al. [88] studied the oil-absorption performance of CPPs and found that when the porosity of CPPs increased from 72 to 91%, the adsorption capacity of toluene increased from 12.5 to 29.5 g/g, a growth rate of 136%.

## 5. Conclusions and Outlook

### 5.1. Conclusions

Traditional oil-absorbing materials such as fibers, aerogels, and resins have the advantages of good oil–water selectivity, high oil-absorption ratio, and fast oil-absorption rate in oil pollution treatment. Inorganic non-metallic materials such as cement-based materials and geopolymers have broad application prospects in dealing with oil pollution.

By adding organic modified materials, the oil-absorption performance of cement-based materials can be improved in terms of lipophilicity, oil-absorption capacity, and oil-absorption rate. Adding 10% Acronal S400F emulsion can reduce the water absorption rate of cement stone by 75% and enhance the oil-absorption rate by 62%; adding 5% polyethylene glycol can increase the oil–water relative permeability of cement stone to a maximum of 1.2.

Based on the research on the oil-absorbing performance of cement-based materials, combined with the research on the oil-absorbing properties, oil-absorbing mechanism, and adsorption model of oil-absorbing materials, the effects of physical and chemical properties such as porosity, specific surface area, pore interface, outer surface, oil-absorption strain, and pore network on the oil-absorption performance of materials are summarized. The influencing factors of oil-absorption performance are summarized from various angles to provide a reference for the design of multifunctional cement-based materials.

### 5.2. Outlook

The pore structure has a great influence on the adsorption performance of oil-absorbing materials. At present, there are many studies on the pore structure of various oil-absorbing materials, but there are relatively few studies on the relationship between the pore structure and oil-adsorption performance of cement-based materials. The research on the lipophilicity and oil absorption of cement-based materials is mainly concentrated in the field of oil well cement research, and the research on the use of its adsorption properties in purifying oil-polluted environments is still to be carried out.

## Figures and Tables

**Table 1:** Synthesis of oil well cement modified by organic additives.

Type	Modified Materials (Mass Percentage)	Advantages	Disadvantages
Lipophilic cement slurry with LQ emulsion [51]	LQ emulsion (15%)	The hydrophobic and lipophilic properties are obvious, the wettability of the oil interface is improved, and the bonded interface strength of the oil interface increases from 0 to 1.44 MPa	Static fluid loss is reduced by 40%, compressive strength is reduced by 28%, thickening time is increased by 88%
Polyvinyl-alcohol-modified cement [52]	Polyvinyl alcohol (1%)	The filtrate of the cement decreases, the bond strength increases, and the fracture performance is increased by 38%	When the dosage reaches 2%, the strength decreases, and the cement hydration time slows down
Silica-modified oil absorbent resin cement [41]	Nano-silica-modified oil-absorbing resin (10%)	The dispersibility and oil-absorption performance of the modified resin increase by 6%, the impact resistance of the cement stone increase by 46%, and the self-healing ability when encountering oil is enhanced	The density and fluidity of cement slurry decrease, the compressive strength decreases by 51%, and the flexural strength decreases by 20%
High-permeability cement [53]	Oil soluble resin (15%)	It has selective permeability for diesel oil, the oil–water permeability ratio reaches 7.1, and the water loss of cement slurry is reduced	The thickening time increases, and the compressive strength decreases by 20%
Selectively permeable cement [54]	Polyethylene glycol (5%)	The oil-wet property on the surface of the cement stone is better than the water-wet property, and the internal pores of the cement stone change to the oil-wet property, and the oil–water permeability ratio reaches 1.2	A small increase in oil phase permeability
Polymer-modified hardened cement [55]	Acronal S400F emulsion (10%)	The linear expansion of the cement stone after absorbing water and oil is significantly increased, which is beneficial to the self-healing of cement stone cracks after absorbing water and oil	Polymer film formation results in a decrease in water absorption rate with a corresponding decrease in permeation rate

**Table 2:** Effect of physicochemical properties of oil-absorbing materials on oil-absorption performance (The number of stars represents the importance of influence).

Physical and Chemical Properties of Oil Absorbing Materials	Definition	Effect on Oil-Absorption Performance	Influence Level
Specific surface area [87]	The total area of a unit mass of material	The high specific surface area is conducive to the adsorption of molecules; however, if the specific surface area is higher than a certain value, the oil absorption decreases, and the oil-absorbing material should take an appropriate specific surface area.	★★★
Porosity [88]	Pore volume as a percentage of the material’s natural state system	The porosity of the material directly affects the oil absorption, and the material with high porosity has more space to absorb oil substances.	★★★★
Pore interface [89]	The pore interface of the pores inside the material	The wettability of the internal pore interface directly affects the oil-absorption rate. The internal pores of normal oil-absorbing materials are all lipophilic, so the strength of lipophilicity does not have a significant impact on the oil absorption.	★
The outer surface of the material [37]	The outermost layer of the oil-absorbing material, the interface that first contacts the oil	The wettability of the outer surface has a great influence on the oil-absorption performance. Hydrophobic and lipophilic treatment is applied to the outer surface of the material with low oil–water selectivity, the adhesion of oil substances on the surface becomes stronger, and it can better diffuse into the interior.	★★★
Oil strain [55]	Deformation of the internal pores of the material after oil absorption due to pore pressure	The deformation of the material usually increases significantly after oil absorption, which leads to an increase in oil absorption and a decrease in oil retention. In order to achieve material characteristics, it is necessary to balance the design of materials with large oil absorption and good oil retention.	★★
Pore network [90]	Network formed by pores and pore channels	Oil substances diffuse into the interior through the pore network, and the liquid phase can diffuse into the pores more fully. The single pore channel or poor connectivity affects the oil-absorption rate and oil absorption.	★★

## Data Availability

The relevant data can be available upon request by contact with the corresponding authors.

## Nomenclature

EP	Epoxy resin
MF	Melamine foam
SMA	Stearyl methacrylate
BMA	Butyl methacrylate
PO	Portland cement
SOO	Superhydrophobic–superoleophilic
Cu	Copper
CCSSM	Cement-coated stainless-steel meshes
PEG	Polyethylene glycol
